# Comparison of mortality and its causes in patients with complicated systemic lupus erythematosus on hemodialysis versus peritoneal dialysis: A meta-analysis

**DOI:** 10.1097/MD.0000000000030090

**Published:** 2022-08-12

**Authors:** Wenjun Gou, Yan Hong Tuo

**Affiliations:** a Department of Nephrology, The First Affiliated Hospital of Yangtze University, Jingzhou, Hubei, People’s Republic of China; b Department of Nephrology, The Central Hospital of Wuhan, Tongji Medical College, Huazhong University of Science and Technology, Wuhan, Hubei, People’s Republic of China.

**Keywords:** cardiovascular death, hemodialysis, infection, mortality, peritoneal dialysis, risk ratios, systemic lupus erythematosus

## Abstract

**Background:**

Lupus nephritis is one of the most serious complications of systemic lupus erythematosus (SLE). Ten percent to 20% of patients with SLE progress to end-stage renal disease and would require renal replacement therapy or renal transplantation. In this analysis, we aimed to systematically compare mortality and the causes of mortality in patients with complicated SLE who were treated on hemodialysis (HD) versus peritoneal dialysis (PD).

**Methods:**

Cochrane Central, Medical Literature Analysis and Retrieval System Online, Google Scholar, Web of Science, Excerpta Medica dataBASE, and http://www.ClinicalTrials.gov were searched for studies that compared HD versus PD in patients with SLE. The RevMan software version 5.4 (RevMan software, Cochrane Collaborations, United Kingdom) was used to analyze data. Heterogeneity was assessed using the Q and the *I*^2^ statistical tests. In this analysis, a random effects model was used during data assessment. Risk ratios (RRs) with 95% confidence intervals (CIs) were used to represent the results following analysis.

**Results:**

A total number of 3405 SLE participants were included in this analysis, whereby 2841 were assigned to HD and 564 participants were assigned to PD. In patients with SLE who were on dialysis, our analysis showed that the risk of mortality was similar with HD and PD (RR, 0.69; 95% CI, 0.45–1.07; *P* = .10). When the cause of mortality was analyzed, cardiovascular death (RR, 0.63; 95% CI, 0.31–1.31; *P* = .22), death due to infection (RR, 0.74; 95% CI, 0.47–1.17; *P* = .20), death due to a respiratory cause (RR, 1.06; 95% CI, 0.18–6.21; *P* = .95), cause of death due to SLE flare up (RR, 2.54; 95% CI, 0.39–16.37; *P* = .33), and other causes of death (RR, 0.79; 95% CI, 0.35–1.77; *P* = .57) were not significantly different with HD and PD.

**Conclusion:**

This current analysis showed that in SLE patients who required dialysis, the risk of mortality between HD and PD was similar, and the causes of death including cardiovascular, infective, respiratory, SLE flare up, and other causes were not significantly different. Therefore, both dialysis methods were tolerable in these patients with SLE. Further studies with larger data would be required to confirm this hypothesis.

## 1. Introduction

Systemic lupus erythematosus (SLE) is a rare autoimmune disorder, which affects a small percentage of patients, especially women.^[1]^ Lupus nephritis (LN) is one of the most serious complications of SLE, and it often results in end-stage renal disease (ESRD).^[2]^ It has been shown that 10% to 20% of SLE patients progress to ESRD and would require renal replacement therapy or renal transplantation.^[3]^ Despite advances in renal replacement therapy, which have reduced the rate of mortality in such patients, the number of patients who would require hemodialysis (HD), peritoneal dialysis (PD), or renal transplantation has increased.

Even though researches in this field are extremely important, only few small cohorts have been carried out to assess the outcomes of HD and PD in these patients with SLE.

We aimed to systematically compare mortality and the causes of mortality in patients with complicated SLE who were treated on HD versus PD.

## 2. Methods

### 2.1. Search databases and search strategies

The following databases including Cochrane Central, Medical Literature Analysis and Retrieval System Online, Google Scholar, Web of Science, Excerpta Medica dataBASE, and http://www.ClinicalTrials.gov were searched for studies that compared HD versus PD in patients with SLE. This search was carried out between June 2021 to September 2021, and it was limited to studies that were published before October 2021.

Reference lists of relevant studies were also checked for any vital study.

The following search terms were used:

1. HD, PD, and systemic lupus erythematous;
2. HD, PD, and SLE;
3. HD, PD, lupus, and mortality;
4. SLE, dialysis, and mortality; and
5. SLE and dialysis.

### 2.2. Inclusion and exclusion criteria

The inclusion criteria were:

1. Studies that compared HD versus PD in patients with SLE;
2. Studies that reported mortality in both the experimental and the control group separately, or the causes of mortality as their endpoints; and
3. Studies that were published in English language.

The exclusion criteria were:

1. Studies that were literature reviews, meta-analyses, systematic reviews, or case studies;
2. Studies that did not report mortality or its causes as their endpoints; and
3. Duplicated studies.

### 2.3. Outcomes

All-cause mortality was considered in this analysis. Causes of mortality, registered in Renal Epidemiology and Information Network by nephrologists according to a common manual, were carefully checked and verified by clinical research assistants.

Table 1 reports the causes of death that were assessed in this analysis comparing SLE patients who required HD or PD.

**Table 1:** Outcomes reported.

Studies	Cause of death	Follow-up time period
Chang et al^[4]^ 2013	Infection, cardiovascular cause, gastrointestinal, pulmonary, malignancy, others	9 yr
Kang et al^[5]^ 2011	Disease flare up, infection, cardiovascular disease, malignancy, bleeding	5 yr
Levy et al^[6]^ 2015	Cardiovascular disease, infections, cachexia, major bleeding, respiratory insufficiency, cancer, hyperkalemia, hypoglycemic coma, others	5 yr
Tsai et al^[7]^ 2019	SLE flare up, infection, cardiovascular cause, malignancy, seizure	10 yr
Weng et al^[8]^ 2009	Cardiovascular cause, pulmonary edema, sepsis	3 yr

The following causes of death were considered as the endpoints:

1. Cardiovascular cause;
2. Infection;
3. Respiratory cause;
4. SLE flare up; and
5. Other causes.

### 2.4. Data extraction and quality assessment

The authors independently extracted data from the selected studies. Several parameters, including the authors’ names, the publication year, the mean age of the participants, the comorbidities, the type of studies, the total number of SLE participants who were assigned to HD and PD, respectively, the time period of patients’ enrollment, the mortality reported in each study, and the causes of death as well as the total number of events associated with each cause were carefully extracted.

Any disagreement that followed was discussed and resolved between the authors.

The quality assessment of the studies was carried out by the Newcastle-Ottawa Scale (NOS),^[9]^ whereby grades were given to rate the risk of bias, grade A representing a low risk, whereas grade C representing a high risk of bias.

### 2.5. Statistical analysis

The RevMan software version 5.4 (RevMan software, Cochrane Collaborations, United Kingdom) was used to analyze the data. Heterogeneity was assessed using the Q statistic test and the *I*^2^ statistic test. An analysis of outcome with a *P* value of less or equal to .05 was considered to be statistically significant, whereas a *P* value above .05 was not significant. For the *I*^2^ statistical assessment, the lower the *I*^2^ value, the lower the heterogeneity, whereas heterogeneity increased with increasing *I*^2^ value. In this analysis, a random effects model was used during data assessment. Risk ratios (RRs) with 95% confidence intervals (CIs) were used to represent the results following analysis. Sensitivity analysis was also carried out. In addition, publication bias was assessed through funnel plots.

### 2.6. Ethical guideline

This study is a meta-analysis whereby an ethical or a board review approval was not required. Data were extracted from previously published original studies.

## 3. Results

### 3.1. Searched outcomes

**Figure 1.**
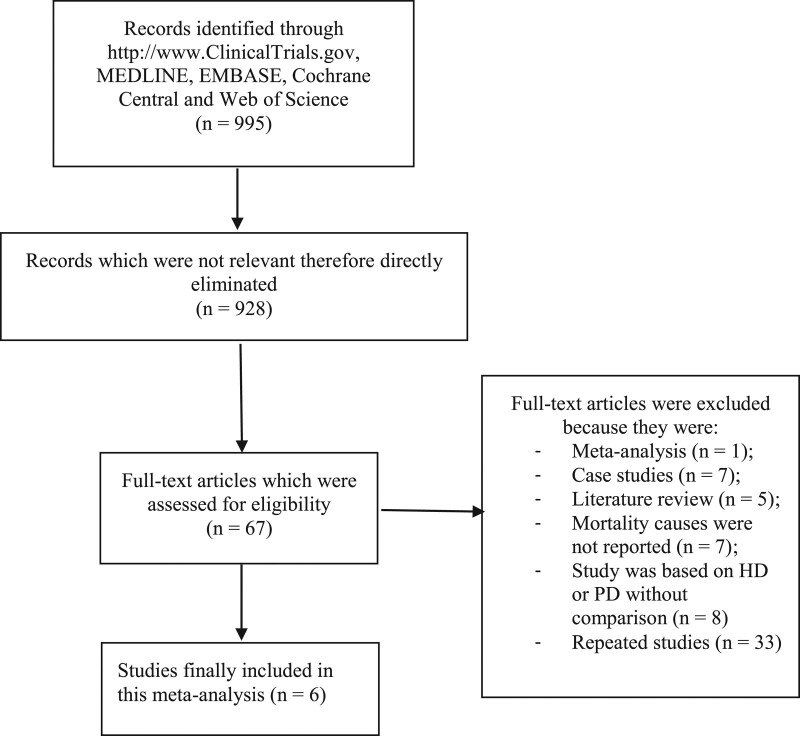
Flow diagram representing the study selection. HD = hemodialysis, PD = peritoneal dialysis.

The Preferred Reporting Items for Systematic Reviews and Meta-Analyses guideline were followed.^[10]^ Our search outcomes resulted in a total number of 995 publications. The authors carefully assessed the titles and abstracts of the papers, and several manuscripts were eliminated due to irrelevance. A total number of 67 full-text publications were assessed for eligibility. Based on the criteria for inclusion and exclusion, further eliminations were carried out. Finally, only 6 publications^[4]^ were selected for this analysis. Figure 1 shows the flow diagram for the study selection.

### 3.2. General features of the studies

All the studies were observational studies. Table 2 lists the general features of the studies. A total number of 3405 SLE participants were included in this analysis, whereby 2841 were assigned to HD and 564 participants were assigned to PD. The time period of patients’ enrollment ranged from year 1990 to 2012 as shown in Table 2.

**Table 2:** Main features of the studies.

Studies	Type of study	Country of origin or region	Time period of patients’ enrollment (yr)	Number of SLE participants undergoing HD (n)	Number of SLE participants undergoing PD (n)
Chang et al^[4]^ 2013	Observational	Taiwan (Asia)	1997–2006	813	260
Kang et al^[5]^ 2011	Observational	Korea (Asia)	1990–2007	28	14
Levy et al^[6]^ 2015	Observational	France (Europe)	2002–2012	308	60
Tsai et al^[7]^ 2019	Observational	Taiwan (Asia)		42	12
Weng et al^[8]^ 2009	Observational	Taiwan (Asia)	1999–2007	14	22
Wu et al^[11]^ 2014	Observational	Taiwan (Asia)	1998–2009	1641	196
Total number of participants (n)				2841	564

### 3.3. Baseline features

Table 3 lists the baseline features of the participants. SLE participants who were assigned to the HD group had a mean age ranging from 35.0 to 48.7 years, whereas those assigned to the PD group had a mean age ranging from 33.2 to 44.8 years. Most of the participants were females. The percentages of patients having comorbidities including high blood pressure, diabetes mellitus, and coronary artery disease were also listed in Table 3. Based on the data shown in Table 3, there were no significant differences in baseline features between participants of the 2 groups.

**Table 3:** Baseline features of the participants.

Studies	Age (yr)	Females (%)	HBP (%)	DM (%)	CAD (%)
	HD/PD	HD/PD	HD/PD	HD/PD	HD/PD
Chang et al^[4]^ 2013	42.6/34.1	80.9/86.2	69.2/76.5	16.1/10.4	14.8/7.70
Kang et al^[5]^ 2011	35.0/41.0	82.1/92.3	—	—	—
Levy et al^[6]^ 2015	44.8/44.8	—	67.9/67.9	9.40/9.40	8.40/8.40
Tsai et al^[7]^ 2019	36.4/33.2	—	—	—	—
Weng et al^[8]^ 2009	48.7/37.6	—	—	—	—
Wu et al^[11]^ 2014	39.3/36.2	84.4/88.8	63.1/76.5	10.9/8.20	13.3/9.70

### 3.4. Main results

**Figure 2.**
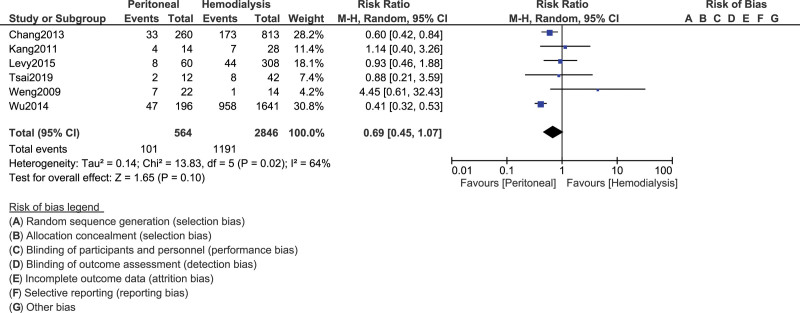
Comparing mortality in SLE patients undergoing HD vs PD. CI = confidence interval, df = degrees of freedom, HD = hemodialysis, PD = peritoneal dialysis, SLE = systemic lupus erythematosus.

In patients with SLE who were on dialysis, our analysis showed that the risk of mortality was similar with either HD or PD (RR, 0.69; 95% CI, 0.45–1.07; *P* = .10), as shown in Figure 2.

**Figure 3.**
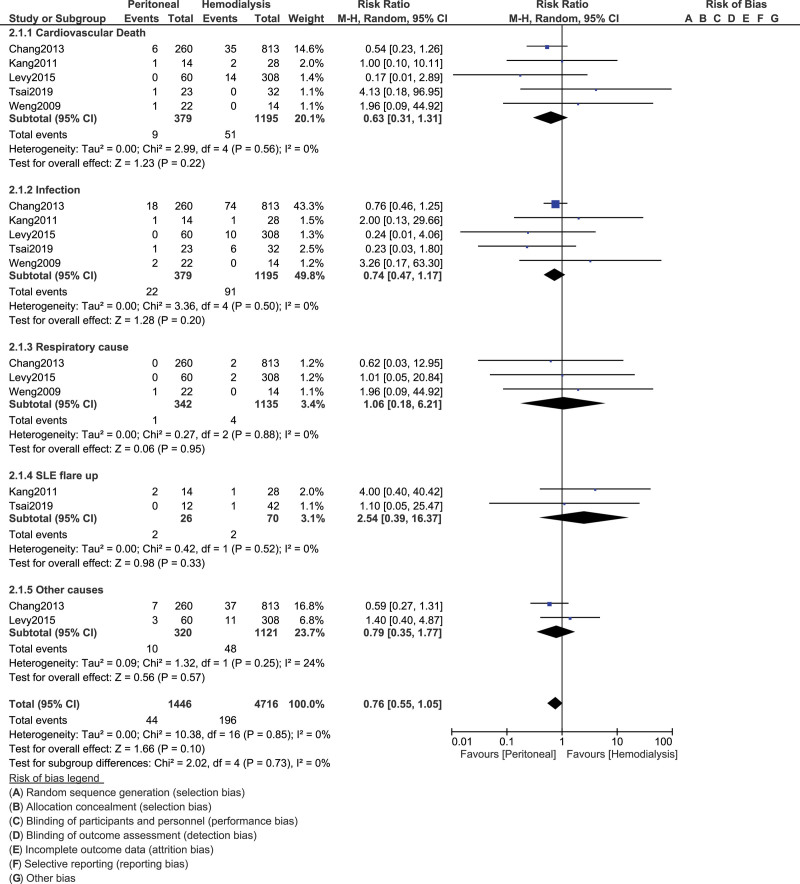
Comparing the causes of mortality in SLE patients undergoing HD vs PD. CI = confidence interval, df = degrees of freedom, HD = hemodialysis, PD = peritoneal dialysis, SLE = systemic lupus erythematosus.

When the causes of mortality were analyzed in those patients with SLE who required dialysis, cardiovascular death (RR, 0.63; 95% CI, 0.31–1.31; *P* = .22), death due to infection (RR, 0.74; 95% CI, 0.47–1.17; *P* = .20), death due to a respiratory cause (RR, 1.06; 95% CI, 0.18–6.21; *P* = .95), cause of death due to SLE flare up (RR, 2.54; 95% CI, 0.39–16.37; *P* = .33), and other causes of death (RR, 0.79; 95% CI, 0.35–1.77; *P* = .57) were not significantly different with HD or PD. In other words, the cause of death was similar in these patients with SLE who required either HD or PD, as shown in Figure 3. The results have been represented in Table 4.

**Table 4:** Results of this analysis.

Endpoints	RR (95% CI)	*P*	*I* ^2^
Mortality	0.69 (0.45–1.07)	.10	64%
Cause of death			
Cardiovascular cause	0.63 (0.31–1.31)	.22	0%
Infection	0.74 (0.47–1.17)	.20	0%
Respiratory cause	1.06 (0.18–6.21)	.95	0%
SLE flare up	2.54 (0.39–16.37)	.33	0%
Others	0.79 (0.35–1.77)	.57	24%

**Figure 4.**
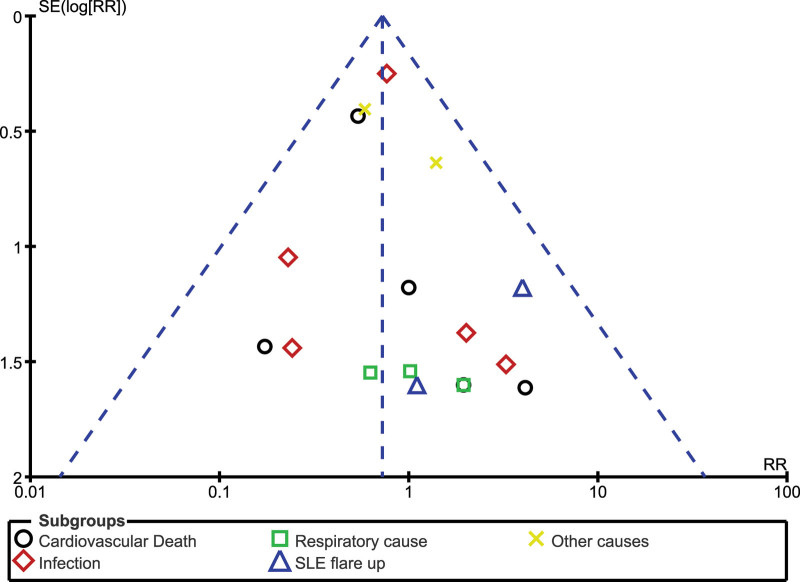
Funnel plot showing publication bias (A). RR = risk ratio, SLE = systemic lupus erythematosus.

**Figure 5.**
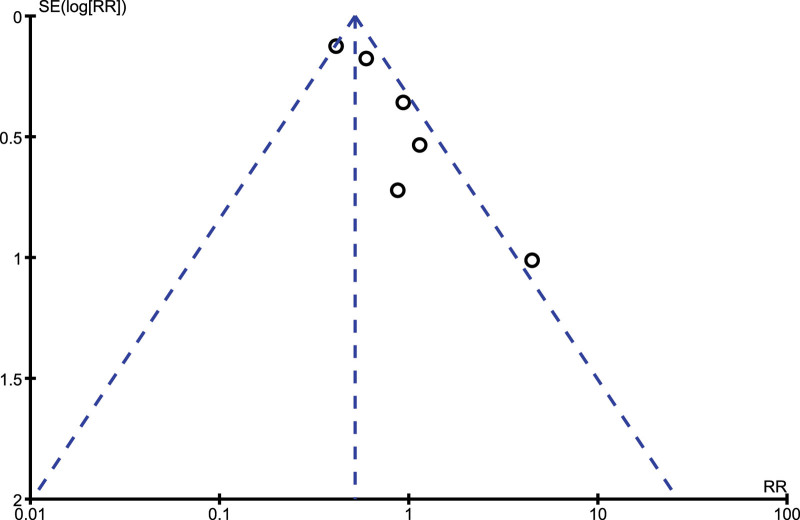
Funnel plot showing publication bias (B). RR = risk ratio.

Sensitivity analysis showed consistent results throughout. An exclusion of each study by turn did not cause any significant change in the main results of this analysis. There was also little evidence of publication bias among the studies that were involved in assessing mortality as well as the causes of death, as shown in Figures 4 and 5.

## 4. Discussion

In this analysis, we aimed to compare mortality and the causes of mortality in SLE patients who were on maintenance dialysis, more specifically on HD and PD. Results of this analysis showed a similar risk of mortality, and the causes of death were not significantly different.

Similar to the results of this analysis, another study including 55 patients with SLE showed that these patients had excellent survival rates with dialysis, and no significant difference in survival or disease activity between those SLE patients who were treated with HD or PD was observed.^[3]^ Another retrospective nationwide population-based study using the National Health Insurance Research Database in Taiwan showed no impact of different dialysis modalities on mortality of the female patients with SLE.^[12]^

However, in a matched case-control study showing the long-term clinical outcomes of LN patients undergoing PD, the authors demonstrated that LN patients undergoing PD had poor patient survival rate and higher infection and hospitalization rates.^[13]^ Another study also showed PD to be associated with a higher risk of infection, and the authors concluded that SLE in itself may compromise the immune system of uremic patients.^[14]^ But, it was also revealed that in SLE patients, PD and HD did not differ in patients and technical survival.^[15]^ In the Dutch Working Party on SLE, the authors stated that patients with SLE had excellent survival with dialysis and that no difference in survival rate was observed between patients undergoing HD or PD.^[3]^

In addition, a cohort study that consisted of 34 SLE ESRD patients and that was published in the year 2016 showed that SLE ESRD patients with antiphospholipid antibodies, more specifically, antiphospholipid/lupus anticoagulant had higher all-cause mortality risk compared with similar patients without those antibodies.^[16]^

Nevertheless, researches in this field should be encouraged, and newer treatment and management techniques, a greater number of participants would be required to further investigate on this particular type of patients.

## 5. Limitations

This study also had limitations. First of all, the total number of participants was low, and this might have affected the final outcomes of this analysis. Furthermore, less studies were published on this topic comparing HD versus PD in patients with SLE, and therefore, only a few studies were available to be included in the data analysis. Important outcomes such as peritonitis and bleeding were reported in only 1 or 2 studies that were less for comparison and had to be neglected. Another limitation was that the duration of disease, the time period since the start of HD or PD, the other comorbidities among the patients were not taken into consideration. In addition, the length of follow-up time period was also not considered. Due to limited data, it was not possible to compare mortality in SLE patients within patients from different ethnical backgrounds. We should also not ignore the fact that all studies that were included in this analysis were observational studies and most of the patients were Asian people. Selection bias could be another limitation of this analysis. Moreover, due to unavailability of data, the evaluation of residual renal function and duration on dialysis modality as well as dialysis vintage on mortality was not possible. In addition, in this meta-analysis, the 2 studies^[4,11]^ encompassed of 85.5% of all the patients, specifically 2910 patients out of total of 3405 patients, and this clearly showed a strong advantage for PD, and even the whole data would indicate a significant advantage for PD if analyzed by a fixed effect model. Thus, heterogeneity among a minority of cases was a main reason for a nonsignificant difference in mortality according to the random effects model. This could be another major limitation of this meta-analysis.

## 6. Conclusion

Our analysis showed that in SLE patients who required dialysis, the risk of mortality between HD and PD was similar, and the causes of death including cardiovascular, infective, respiratory, SLE flare up, and other causes were not significantly different between HD and PD. Therefore, both dialysis methods were tolerable in these patients with SLE. Further studies with larger data and further debates would be required to confirm this hypothesis.

## Author contributions

The authors, Drs Wenjun Gou and Yan Hong Tuo, were responsible for the conception and design, acquisition of data, analysis and interpretation of data, drafting the initial article, and revising it critically for important intellectual content. All the authors agreed and approved the article as it is. Dr Wenjun Gou is the first author.
